# Two pediatric cases of pine nut allergy proved by oral food challenge

**DOI:** 10.1186/2045-7022-3-S3-P154

**Published:** 2013-07-25

**Authors:** J Ponsaille, T Bourrier

**Affiliations:** 1 Private practice; 2 Pediatric Pneumology and Allergy Department, GCS Hôpitaux Pédiatriques de Nice CHU Lenval, Nice, France

## Background

Allergy to pine nuts is uncommon. Cases are reported in the literature but an oral challenge is rarely described especially in children.

## Methods

We report the observations of two boys of age 5 and 11 formerly diagnosed with common food allergens. A secondary sensitization to pine nuts appears in skin prick tests using a commercial extract **(**Stallergènes^®^, Antony, France**)**. Specific serum IgE test (CAP-FEIA f253 Phadia^®^) were performed. Because it was impossible to precise the consumption of pine nuts (anamnesis not informative enough), an oral food challenge was performed confirming.

## Results

Skin prick tests were positive (8 and 20 mm respectively). Specific IgE tests were positive (4.54 kU/l and 1.75 kU/l respectively). In both cases, oral food challenge confirm the allergy to pine nuts (eliciting dose 1000 mg and 500 mg respectively). Allergic reaction was controlled by medical team without anaphylaxis ; although such reaction is described in the literature with pine nuts. However, as reported in the literature, our patients had other food sensitizations. Indeed, cross reactivity is reported for food allergens (almond, peanuts and nuts in general) and also for respiratory allergens (pine pollen and Artemisia).

Exploration of pine nuts allergy should be careful because of a risk of anaphylaxis with native extracts for prick tests: therefore, commercial extracts should be used first.

## Conclusion

Pine nut is an increasing allergen because of its wide availability and because it is often hidden. The diagnosis is based on skin prick tests and specific serum IgE test when linked to a suggestive history. Oral challenge may be necessary to prove the allergy, especially to an unnecessary diet (i.e. exclusion of pine nuts).

## Disclosure of interest

None declared.

